# Current Approach and Future Directions in the Diagnosis and Prognosis of Keratinocyte Carcinomas

**DOI:** 10.3390/jcm12123974

**Published:** 2023-06-11

**Authors:** Cristian Scheau, Constantin Caruntu, Ana Caruntu

**Affiliations:** 1 Department of Physiology, The Carol Davila University of Medicine and Pharmacy, 050474 Bucharest, Romania; cristian.scheau@umfcd.ro; 2 Department of Radiology and Medical Imaging, “Foisor” Clinical Hospital of Orthopaedics, Traumatology and Osteoarticular TB, 021382 Bucharest, Romania; 3 Department of Dermatology, “Prof. N.C. Paulescu” National Institute of Diabetes, Nutrition and Metabolic Diseases, 011233 Bucharest, Romania; 4 Department of Oral and Maxillofacial Surgery, “Carol Davila” Central Military Emergency Hospital, 010825 Bucharest, Romania; ana.caruntu@gmail.com; 5 Department of Oral and Maxillofacial Surgery, Faculty of Dental Medicine, Titu Maiorescu University, 031593 Bucharest, Romania

Keratinocyte carcinomas (KCs) are malignancies developed from keratinocytes or their precursors. They are comprised of squamous cell carcinomas (SCCs) and basal cell carcinomas (BCCs), and represent the most frequent type of cancer in the Western world [1]. A particular feature of this group of diseases is the multitude of environmental and genetic risk factors potentially involved in carcinogenesis, some clearly established while others still under investigation [2]. Moreover, current research is increasingly focused on developing highly efficient diagnostic and therapeutic strategies in the management of this pathology.

Consistent efforts are invested in the identification of a panel of specific biomarkers for the early diagnosis of SCCs. In their paper, Dikova et al. tested a set of salivary cytokines in oral SCC (OSCC) patients and showed that IL-6, IL-8, TNF-α, and other serum factors differed significantly from the control group. In addition, IL-6 and TNF-α were higher in more advanced stages of the disease [3]. These findings are in accordance with other recent papers, such as the work of Babiuch et al. who detected increased values of the abovementioned cytokines in both tissue specimens and saliva of patients with OSCC but also with oral potentially malignant disorders [4]; the authors, therefore, showed that variation in specific cytokine levels may indicate malignant transformation, pointing towards IL-8 as the most significant biomarker in the tissue samples. Extending the study of cytokines as biomarkers for KCs, Caruntu et al. sought a larger panel of cytokines in the sera of OSCC patients and showed that interleukins 1β, 6, 8, 10, and TNF-α are increased before treatment compared to healthy controls and that their levels may not normalize after completion of treatment. The authors suggested that systemic factors are also involved in modulating the cytokine levels, implying complex interferences between regulation pathways [5]. Moreover, the same research team has tested the value of final catabolism products in the diagnosis and assessment of the tumoral behavior of OSCC [6]. The authors showed that urea levels are decreased in patients with advanced disease and that levels correlate with the presence of lymph node involvement. This simple and accessible test is not only a useful finding in regard to positive diagnosis but can also provide information related to tumor protein catabolism dysregulation and potential development of aggressive features in OSCC.

Another diagnostic approach that was recently considered for the early diagnosis of KCs and premalignant conditions such as actinic cheilitis (AC) is reflectance confocal microscopy (RCM). This non-invasive method of skin imaging has emerged as a reliable in vivo diagnostic technique, especially useful in areas of the skin with a thinner epithelium, such as the lip. In their paper, Lupu et al. demonstrated specific features of SCC such as the destruction of the epidermal architecture and inflammation of the dermis [7]. Furthermore, they noted RCM characteristics of AC including hyper- and parakeratosis, as well as dyskeratosic epidermal cells and were able to measure blood vessel diameter and density, showing that RCM is a useful method in discriminating between SCC and its precursor condition, AC. Consequently, Shavlokhova et al. assessed the value of ex vivo fluorescent confocal microscopy (FCM) as an alternative diagnostic method to classic histopathology in OSCC [8]. The authors used deep learning to train a convolutional neural network (CNN) to classify cancer tissue in OSCCs and validated their machine learning model, therefore encouraging further work on the use of alternative diagnostic methods for KC.

Another recent study has applied the use of CNNs in the diagnosis of KC, combined with a vector machine. Courtenay et al. used hyperspectral imaging for the non-invasive diagnostic of BCC [9]. They showed that machine learning can have an accuracy of up to 90% in correctly diagnosing BCC, specifically in the range from 573.45 to 779.88 nm. The authors further proposed that the technique may be expanded to the detection of SCC, if the appropriate spectrum is applied. In a more recent paper, Wang et al. applied multispectral imaging for the diagnosis of cutaneous SCC and showed that the method can provide both qualitative and quantitative data [10]. The authors evaluated the nucleocytoplasmic ratio in the normal skin cells versus SCC lesions and quantified cell nuclei, collagen, and keratin pearls. Their encouraging results have opened a new direction in the research for accessible and affordable diagnostic methods that also provide histological data. Interestingly, Lindholm et al. developed a hand-held 3D spectral imager that used wavelengths between 477 and 891 nm and CNNs to accurately differentiate between malignant and benign skin lesions which also works on complex skin surfaces [11].

A closer look at the pathogenesis of KC may prove useful in understanding the evolution of this group of diseases and provide insight into developing further diagnostic instruments. Shieh et al. showed that salivary lncRNA XIST expression was lacking in patients with OSCC, therefore implying it as a risk factor for OSCC [12]. The authors went further and proposed salivary lncRNA XIST as a biomarker that would predict the risk of developing OSCC. On a related topic, in their comprehensive study, Kirtane et al. assessed the genomic mechanisms that facilitate immune evasion and may play a prognostic role [13]. The authors concluded that immune evasion takes place as a consequence of accumulated genetic mutations that combine the detrimental effects of losing tumor-suppressing activity and amplifying oncogenes. Furthermore, the paper proposes the future perspective of reversing immune evasion via epigenetic targeted therapies of even exploiting genomic events, which may prove essential in improving prognosis and survival in these patients. Chaudhury et al. showed that measuring the expression of cancer stem cells in tongue SCC can yield relevant diagnostic data in terms of tumor size, histopathological grading, cancer dissemination, and mortality [14]. Additionally, in another recent paper, more attention was brought to the understanding of cellular signaling pathways involved in the carcinogenetic process of BCC. In their paper, Tampa et al. emphasized the importance of the Hedgehog (Hh) pathway regulation by the PTCH1 protein, which shows altered levels through mutations in its gene, therefore triggering multiple BCCs [15]. The authors also present the crosstalk between the Hh pathway and other constitutive signaling pathways such as PI3K/AKT/mTOR, Wnt/β-catenin, and Notch pathways and conclude that a deeper understanding of the interactions between the signaling pathways in BCC can be of paramount importance in the early diagnosis and development of new treatment options for these patients.

In conclusion, considering all recent advancements in the diagnostic and prognosis of KCs, doubled by the progress in the understanding of specific processes occurring in the pathogenesis of this group of diseases, we can expect an increase in the development rate of new and improved, probably computer-assisted, technologies with higher accessibility and accuracy, that will increase our chances in fighting the most frequent cancer in the world.
